# Evaluation of Osteoconductive and Antimicrobial Properties of Novel Graphene on Dental Implants: An In Vitro Study

**DOI:** 10.7759/cureus.54172

**Published:** 2024-02-14

**Authors:** Sounyala Rayannavar, Sunil Kumar MV, Mahantesh Bembalgi, Swapnil Shankargouda, Aditya Acharya, Mallikarjun Doddamani, Vinuta Hiremath, Mehul A Shah

**Affiliations:** 1 Department of Prosthodontics Crown and Bridge, KLE Vishwanath Katti Institute of Dental Sciences, KLE Academy of Higher Education and Research, Belagavi, IND; 2 Department of Prosthodontics Crown and Bridge, Jaipur Dental College, Maharaj Vinayak Global University, Jaipur, IND; 3 Department of Prosthodontics Crown and Bridge, Private Practitioner, Belagavi, IND; 4 Research scholar, Department of Public Health Dentistry, KLE Vishwanath Katti Institute of Dental Sciences, KLE Academy of Higher Education and Research, Belagavi, IND

**Keywords:** surface modification, implant osseointegration, implant coating, graphene oxide, bacterial strains

## Abstract

Introduction

Graphene oxide (GO) has emerged as a promising material in dentistry, leveraging its exceptional properties. This study evaluates the physicochemical attributes of GO and elucidates its derived biological properties. These encompass biocompatibility, antibacterial efficacy, as well as its influence on osteogenic and odontogenic differentiation processes. Understanding the intricate interplay between the physicochemical and biological aspects of GO provides valuable insights into its potential applications in various dental contexts.

Materials and methods

The study group (so; titanium discs surface coated with GO) and the control group (co; plain/uncoated machined titanium discs) were divided based on cell attachment and cell proliferation assays (n=60). These groups were further divided into subgroups (n=30) based on the tested time intervals, specifically 24 hours, 48 hours, and 72 hours. The study and controlgroups were further subdivided into three subgroups (n=10) based on the microorganisms tested i.e *Porphyromonas gingivalis, Prevotella intermedia *and* Fusobacteria nucleatum.*

Results

The results of this in vitro study suggest that GO-coated titanium dental implants have both increased osteogenic potential and antimicrobial efficacy. Graphene has good potential as a promising alternative to traditional surface treatments, and a graphene-coated implant can be used for enhanced osseointegration.

Conclusion

The osteogenic potential and the cell attachment were higher on titanium surfaces coated with GO nanoparticles when compared to plain titanium discs at 24, 48 and 72 hours respectively.

## Introduction

The design and implementation of dental implants encounter challenges related to oral biofilm formation and the aggregation of primary etiological agents on the implant surface. Researchers have explored various implant surface coatings with antibacterial properties to address this issue. These coatings aim to reduce the risk of biofilm formation and microbial aggregation, thereby contributing to the long-term success of dental implants [1,2]. Despite being designed as a substitute for natural teeth, dental implants occasionally face failure due to peri-implant mucositis, a biofilm-induced inflammation that can lead to bone loss and result in peri-implantitis [3-5].

Developing an ideal dental implant that simultaneously promotes osseointegration and inhibits bacterial biofilm formation is a challenging task. Surface treatment plays a crucial role in controlling the structural properties of dental implants. Various commercial dental implants have been created with different surface modifications, such as plasma spraying, resorbable blasted media, acid etching, and oxidation, as essential processes to enhance their performance [6].

The field of graphene-based engineered nanomaterials in dentistry, whether utilized as nanomedicines or dental materials, is rapidly expanding [6]. Current research indicates that coating dental implants with graphene and related materials, such as graphene oxide (GO) and reduced graphene oxide (rGO), holds promise for enhancing osteointegration [7]. Various deposition methods for GO coatings are employed, including electrochemical deposition, photo coupling chemistry, and the chemical assembly technique.

Notably, the chemical assembly method is recognized for its ability to uniformly distribute GO coatings on implant surfaces of any shape and structure, presenting a simple and practical treatment approach [8,9]. The highlight of this in vitro study is evaluation of osteoconductive and antimicrobial properties of novel graphene on dental implants.

## Materials and methods

The present in vitro study was conducted at the Central Research Laboratory, Maratha Mandal's NGH Institute of Dental Sciences & Research Centre, Belagavi, Karnataka, and Shivaji University, Kolhapur, Maharashtra. Only identical titanium disc-shaped specimens measuring 10 mm in diameter and 2 mm in thickness were included in the study. Specimens with internal and external porosities and with surface irregularities (Ra> 5µm) were excluded from the study.

Comparison of the study group and control group with Colony Forming Units (CFU) counts, concerning zone of Inhibition in three organisms was done by independent t-test. Comparison of two main groups and three sub-groups with mean of log cell attachment (cells) by two-way Analysis of Variance (ANOVA). Pair-wise comparison of three subgroups with mean of log cell attachment (cells) by Tukeys multiple posthoc procedures.

This research was intended to evaluate osteogenic potential, osteogenic differentiation of graphene oxide-coated titanium discs, and antimicrobial efficacy of *Porphyromonas gingivalis, Prevotella intermedia, *and* Fusobacterium nucleatum* on graphene-coated titanium discs. For osteogenic potential, 120 titanium discs were used which were divided into the Study Group (30) and the Control Group (30). Study Group (so) had titanium discs surface coated with GO and Control Group (co) had plain/uncoated machined titanium discs.

The study and control groups were further divided based on cell attachment and cell proliferation assays (n=60). These groups were further divided into subgroups (n=30) based on the tested time intervals, specifically 24 hours (n=10), 48 hours(n=10), and 72 hours (n=10). The study and control groups were further subdivided into three subgroups (n=1) based on the microorganisms tested i.e. *Porphyromonas gingivalis, Prevotella intermedia *and *Fusobacteria nucleatum.*

The specimen preparation involved polishing, sandblasting with alumina, and ultrasonic cleaning of identical titanium discs. Surface analysis using a profilometer included quantitative and qualitative evaluations on 240 specimens. The quantitative analysis utilized a Contact Stylus Profilometer, determining the average roughness profile (Ra).

The assessment of antimicrobial efficacy in this study included determining the Minimum Inhibitory Concentration (MIC) and Minimum Bactericidal Concentrations (MBC) for graphene oxide against *Porphyromonas gingivalis, Prevotella intermedia, *and* Fusobacterium nucleatum. *Cytotoxicity evaluation was conducted using a 3-[4,5-dimethylthiazol-2-yl]-2,5 diphenyl tetrazolium bromide (MTT) assay.

**Figure 1:**
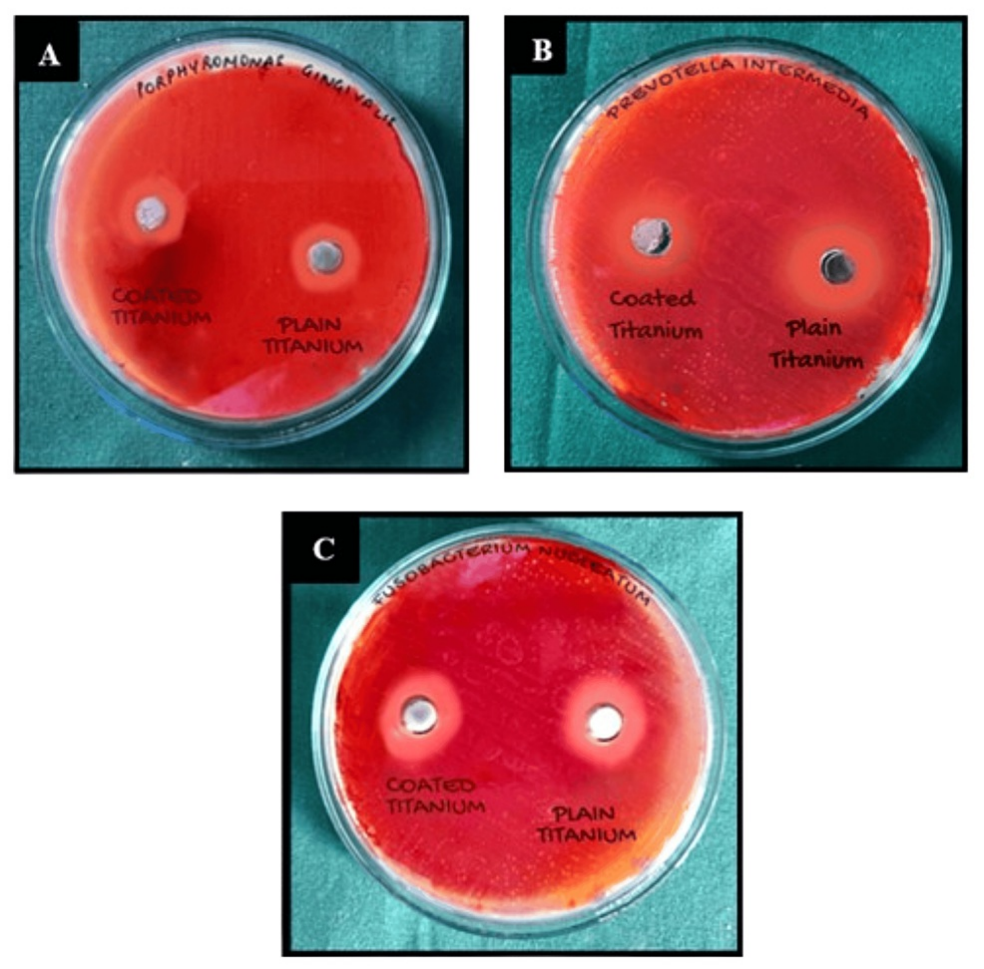
Zone of inhibition against peri-implant pathogens; A: Porphyromonas gingivalis B: Prevotella intermedia C: Fusobacteria nucleatum.

For osteogenic potential assessment, 120 titanium discs were characterized, with 60 coated with graphene oxide. Cell attachment and proliferation assays were performed using MG-63 cell lines at different time intervals. The osteogenic differentiation was appraised using Alizarin Red staining and spectrophotometry at 24, 48, and 72 hours. Microbial analysis utilized the disc-diffusion method against standard bacterial strains *(Porphyromonas gingivalis ATCC 33277, Prevotella intermedia ATCC 25611, *and* Fusobacterium nucleatum ATCC 25586). *The study and control groups were assessed for their effectiveness through zone of inhibition measurements (Figure 1).

**Figure 2:**
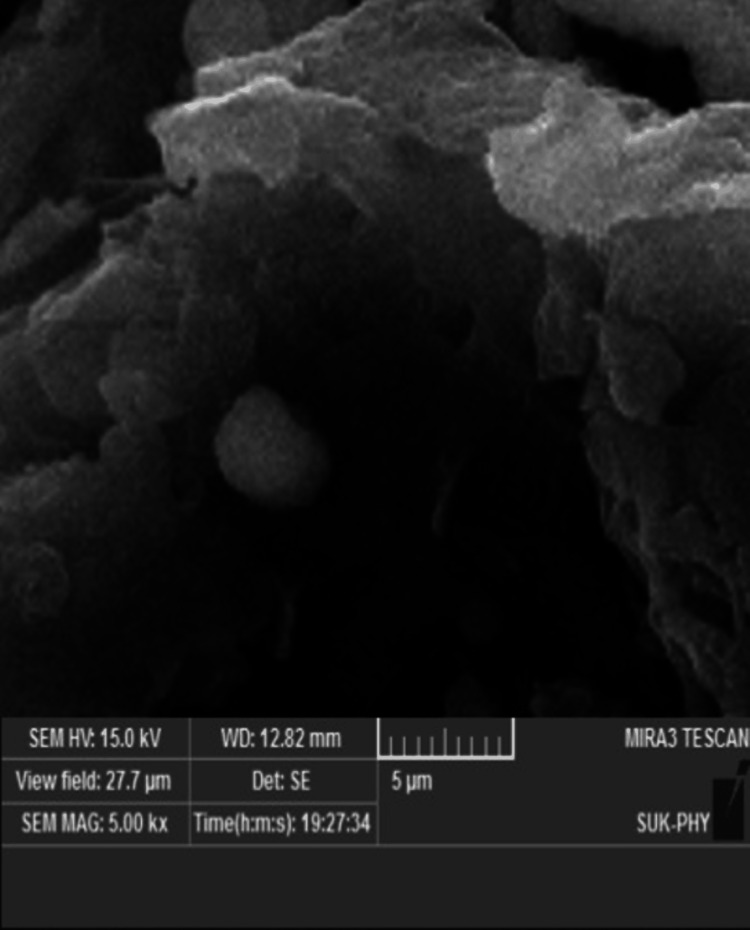
FE-SEM was performed to see the differentiation of the osteoblasts. FE-SEM: Field emission scanning electron microscopy Model: mira3 lmh; Tescan, Brno, Czech Republic

This comprehensive study offers insights into various aspects of GO-coated titanium discs, including surface characteristics, antimicrobial properties, cytotoxicity, osteogenic potential, and microbial analysis. The examination of these factors provides a thorough understanding of how the GO coating influences the surface properties, antimicrobial efficacy, biological response, and microbial interactions of the titanium discs. Qualitative analysis was performed through Field Emission Scanning Electron Microscopy (FE-SEM) at various magnifications, complementing the quantitative results (Figure 2).

## Results

In this study, the osteogenic potential, osteogenic differentiation, and antimicrobial efficacy of GO titanium discs were assessed. The investigation focused on standard bacterial strains, including *Porphyromonas gingivalis, Prevotella intermedia, *and *Fusobacterium nucleatum* (Table 1).

**Table 1:** Comparison of study group and control group with CFU counts, with respect to zone of Inhibition in three organisms by independent t test. CFU: colony-forming unit

Organisms	Groups	n	Mean	SD	SE	t-value	P-value
Porphyromonas gingivalis	Study group	10	13.60	1.06	0.27	-5.4793	0.0001*
	Control group	10	17.87	2.83	0.73		
Prevotella intermedia	Study group	10	15.53	1.60	0.41	-5.0687	0.0001*
	Control group	10	18.00	1.00	0.26		
Fusobacteria nucleatum	Study group	10	13.87	0.92	0.24	-9.2464	0.0001*
	Control group	10	16.53	0.64	0.17		

**Figure 3:**
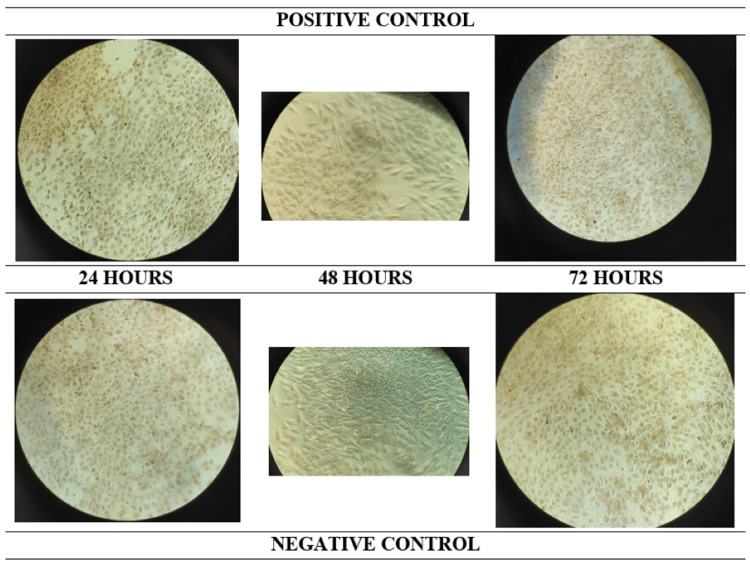
Alizarin red assay to evaluate cell differentiation.

The evaluation involved assessing the cell attachment and proliferation of MG-63 cells on graphene-coated surfaces at 24, 48, and 72-hour intervals. The antimicrobial efficacy was assessed by the disc diffusion method and for osteogenic potential, the cell attachment was assessed using a hemacytometer while cell proliferation was assessed by MTT Assay at 24, 48, and 72 hours. Osteogenic differentiation and mineralization were assessed by Alizarin Red S staining (Figure 3).

**Figure 4:**
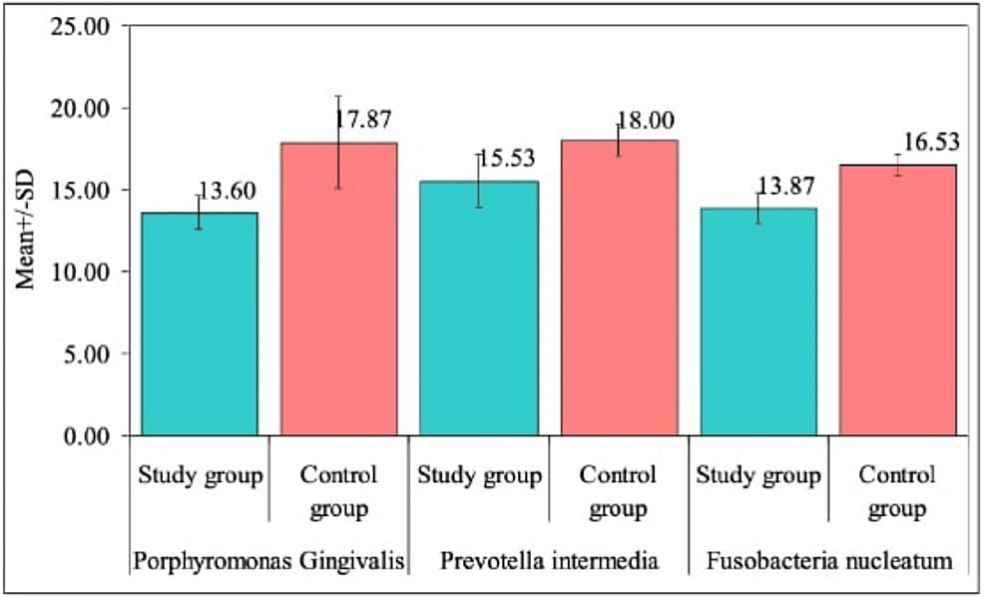
Comparison of study group and control group with colony forming units (CFU) counts with respect to zone of inhibition in three organisms

A significant difference was observed between the study group and the control group with respect to CFU counts in three organisms i.e. *Porphyromonas gingivalis, Prevotella intermedia *and* Fusobacteria nucleatum *at 5% level of significance. It means that the mean CFU counts are significantly higher in control group as compared to study group in all three organisms, which means the zone of inhibition was lower in study group. The findings suggest that the graphene-coated titanium discs exhibited a notable reduction in bacterial counts, indicating potential antimicrobial effectiveness against the specified organisms (Figure 4).

As elicited in Table 2, the main effect of two main groups (study group and control group) on mean of log cell attachment (cells), the calculated value of F(1, 59) is 1331.7789 with p=0.0001. The F-table value of F(1, 59) is 4.0000. The calculated value of F(1, 59) is greater than the F-table value of F(1, 59) i.e. 12.6333>4.0000. It means that the mean of log cell attachment (cells) is different in the study group and control group. The overall effect of three subgroups (24hrs, 48hrs and 72hrs) on the mean of log cell attachment (cells), the calculated value of F(2, 59) is 2.3849 with p=0.1017. The F-table value of F(2, 59) is 3.1500. The calculated value of F(2, 59) is smaller than the F-table value of F(2, 59) i.e. 2.3849<3.1500. It means that the mean of log cell attachment (cells) are similar in 24, 48 and 72 hours respectively.

**Table 2:** Comparison of two main groups and three subgroups with mean of log cell attachment (cells) by two-way ANOVA

Sources of variation	Sum of squares	Degrees of freedom	Mean sum of squares	value	p-value
Main effects					
Main groups (study group and control group)	6.13	1	6.13	113.7789	0.0001*
Subgroups (based on duration of tested time intervals into 24hr, 48hr and 72hr)	0.02	2	0.01	2.3849	0.1017
Two way interaction effects					
Main X sub groups	0.15	2	0.08	16.7734	0.0001*
Error	0.25	54	0.00		
Total	6.55	59			

The interaction effect of two main groups (study group and control group) and three subgroups (24hrs, 48hrs and 72hrs) on mean of log cell attachment (cells), the calculated value of F(2, 59) is 16.7734 with p=0.1477. The F-table value of F(2, 59) is 3.1500. The calculated value of F(2, 59) is smaller than the F-table value of F(2, 59) i.e. 16.7734>3.1500. No significant difference was observed between the three subgroups with mean of log cell attachment (cells) at a significance level of 5% (p > 0.05) (Table 3).

**Table 3:** Tukey's multiple post hoc analysis for mean log cell attachment (cells) in three subgroups

Subgroups	24hrs	48hrs	72hrs
Mean	5.0303	5.0723	5.0691
SD	0.2748	0.3376	0.3926
24hrs	-	-	-
48hrs	P=0.1318	-	
72hrs	P=0.1753	P=0.9879	-

## Discussion

Titanium dental implants have a great success rate. Quick and steady osseointegration on bone-implant contact (BIC) is meant for any effective integration of implants [10,11]. Surface treatments are broadly used to improve a dental implant, with physical and chemical methods [12]. It offers a conducive environment for enhancing both the quantity and quality of osseointegration. Titanium and its alloys are known for their bio-inert properties as it is challenging to bind to bone after placement of implant because of absence of osteoconductive and osteoinductive properties [10-15].

The implant surface is in direct contact with the host tissue, such as bone tissue. Hence, the surface properties are mainly determining factor for the following complex cell behaviour at the bone-implant interface in vivo as well as for the cell response in vitro. Various parameters, for example surface topography, charge and culture conditions (in vitro) or physiological environment (in vivo), chemistry, impact the discrete interactions between cells and the biomaterial. Interestingly, the same basic substrate can aggravate different cell responses when exhibiting different nanostructures.

Graphene-based materials have aroused great attention in the field of bone grafting and implant surface modification due to the unique properties of GO. Its potential for improving osteogenesis has been explored in vitro and in vivo. As a major derivative of graphene, GO has some differences in oxygen content, film diameter, and number of layers according to the production methods involved. Former research indicates that oxygen content, diameter and thickness of nanosheets and post-treatment of graphene-based materials have different effects on osteo-inductive potential.

In a study conducted by Yadav et al., the effect of two GO-coated surfaces created through two distinct methods were studied, further highlighting the influence of the preparation method. One method created a rougher surface, with a non-uniform thickness, where GO was carboxylic rich and displayed an increased inhibitory effect against *Staphylococcus aureus*, while the other method created a smoother surface, where GO presented more epoxy and hydroxyl groups and showed a more selective inhibiting effect against Escherichia coli [16].

In this study, the osteogenic potential, osteogenic differentiation, and antimicrobial effectiveness of graphene-coated titanium discs were investigated against standard bacterial strains, including *Porphyromonas gingivalis, Prevotella intermedia, *and *Fusobacteria nucleatum.* The assessment involved analyzing MG-63 cell attachment and proliferation on graphene-coated surfaces at 24, 48, and 72-hour intervals. Antimicrobial efficacy was determined using the disc diffusion method, while osteogenic potential was evaluated through cell attachment using a hemacytometer and cell proliferation via the MTT assay at 24, 48, and 72 hours. Osteogenic differentiation and mineralization were further examined through Alizarin Red S staining.

One of our study's drawbacks was that it was conducted in vitro; in vivo research can be done in the future. The other limitations include that the cell lines used for this study were MG-63 cells (osteoblasts-like cells) which is an osteosarcoma cell line. It lacks coherence in the capacity of cell differentiation and the method of coating was done with the droplet method. There could be variation in the uniform thickness of the surface of the substrate.

## Conclusions

An increase in life expectancy, as well as the ongoing concern about physical appearance, have elevated the relevance of dental implantology in recent decades. The use of dental implants because of their high predictability of success, represents a reliable treatment for replacement of the missing teeth. The gold standard in implant dentistry is titanium. It has the longest record of predictable clinical performance. The implant surfaces are expected to both enhance the growth of living cells and simultaneously inhibit bacteria. The degree of osseointegration becomes highly dependent on the surface properties of implant materials indicating the modification of implant surface as an important part of surface area for cell attachment. There has been a tremendous advancement in biomaterials in the last decades that has led to the discovery of novel-based materials, devices, nanomaterials design, synthesis, and characterization. Among these, graphene nanomaterials have emerged as a promising material. It has excellent physical, chemical, and mechanical properties and has been indicated to accelerate the growth, proliferation, and differentiation of cells. There have been very few studies on the biomedical applications of graphene nanomaterials, osteogenic potential, and antimicrobial properties in the last few years. hence our study results would be a reliable source to carry out in vivo human studies in the future.
